# Resveratrol Attenuates Fibrosis and Alters Signaling Pathways in Diabetic Cardiac and Skeletal Muscles and Adipose Tissue Without Reversing Structural Damage

**DOI:** 10.3390/ijms26041672

**Published:** 2025-02-15

**Authors:** Célia Maria Cássaro Strunz, Alessandra Roggerio, Paula Lázara Cruz, Luiz Alberto Benvenuti, Maria Cláudia Irigoyen, Antonio de Padua Mansur

**Affiliations:** 1 Laboratório de Análises Clínicas, Instituto do Coracao (InCor), Hospital das Clinicas HCFMUSP, Faculdade de Medicina, Universidade de Sao Paulo, Sao Paulo 05403-900, SP, Brazil; alessandra.roggerio@incor.usp.br (A.R.); maria.irigoyen@incor.usp.br (M.C.I.); 2 Laboratório de Hipertensão Experimental, Instituto do Coracao (InCor), Hospital das Clinicas HCFMUSP, Faculdade de Medicina, Universidade de Sao Paulo, Sao Paulo 05403-900, SP, Brazil; paulalcruz@gmail.com; 3 Laboratório de Anatomia Patológica, Instituto do Coracao (InCor), Hospital das Clinicas HCFMUSP, Faculdade de Medicina, Universidade de Sao Paulo, Sao Paulo 05403-900, SP, Brazil; anpluiz@incor.usp.br; 4 Serviço de Prevenção, Cardiopatia na Mulher e Reabilitação Cardiovascular, Instituto do Coracao (InCor), Hospital das Clinicas HCFMUSP, Faculdade de Medicina, Universidade de Sao Paulo, Sao Paulo 05403-900, SP, Brazil; apmansur@usp.br

**Keywords:** resveratrol, diabetic cardiomyopathy, skeletal muscle atrophy, adipose tissue remodeling, inflammatory pathways, fibrosis

## Abstract

Resveratrol (RSV) improves metabolic functions, but its tissue-specific effects on diabetes remain unclear. This study investigated RSV’s impact on molecular pathways in an experimental model of diabetes in cardiac and skeletal muscles and adipose tissue. Wistar rats were assigned to control (C), control treated with RSV (RC), diabetic (D), and diabetic treated with RSV (RD). Diabetes was induced using streptozotocin and nicotinamide, and RSV was administered for six weeks. In diabetic rats, RSV treatment significantly reduced collagen accumulation in cardiac and skeletal muscle tissues compared to untreated diabetic controls, although it did not restore muscle mass. Adipose tissue in diabetic rats exhibited a significant reduction of 3.4 times in collagen levels following RSV treatment. However, this reduction was not associated with any measurable improvement in tissue function. In cardiac tissue, RSV downregulated phosphorylated protein kinase B (AKT)/AKT and phosphorylated ribosomal protein S6 (rpS6)/rpS6 while mammalian target of rapamycin (mTOR) activity remained unchanged. In skeletal muscle, RSV suppressed rpS6 phosphorylation without affecting (mTOR) signaling. RSV enhanced mTOR and Beclin-1 expression in adipose tissue, though metabolic dysfunction persisted. RSV reduced receptors for advanced glycation end-product expression in all tissues, indicating the modulation of hyperglycemia-driven pathways. RSV improved fibrosis and signaling pathways but failed to reverse abnormal tissue growth patterns, including cardiac hypertrophy, skeletal muscle atrophy, and adipose tissue atrophy.

## 1. Introduction

Diabetes mellitus is a chronic metabolic disorder often accompanied by secondary complications stemming from hyperglycemia, which significantly impacts both the microvascular and macrovascular systems. These complications can affect multiple organs, including the eyes, kidneys, brain, heart, and skeletal muscles [1]. One of the most concerning consequences of diabetes mellitus is the heightened susceptibility to cardiovascular diseases, with diabetic cardiomyopathy characterized by diastolic dysfunction, cardiomyocyte hypertrophy, fibrosis, and metabolic dysregulation [2]. Beyond the heart, these pathophysiological changes extend to metabolic disturbances and structural alterations in skeletal muscle and adipose tissue, exacerbating systemic organ dysfunction [3,4]. The dysregulation of cardiomyocyte function in chronic conditions such as diabetes is primarily driven by alterations in the expression and post-translational modification of key proteins within the cardiomyocytes [5]. This systemic disruption manifests as an imbalance between the synthesis and degradation of contractile proteins. In diabetes, this imbalance leads to cardiac hypertrophy due to increased protein synthesis, while skeletal muscle atrophy occurs due to accelerated proteolysis [6]. The insulin-like growth factor-1 (IGF-1)/protein kinase B (AKT)/mammalian target of rapamycin (mTOR) signaling pathway is central to the regulation of protein synthesis and degradation. This pathway is activated by binding insulin and growth factors to receptors on muscle cell membranes. AKT plays a key role in activating mTOR, a critical regulator of protein synthesis, by promoting ribosomal biogenesis through its downstream effector, S6 kinase 1 (S6K) [7,8]. Moreover, the AKT pathway inhibits protein degradation by modulating the ubiquitin–proteasome and lysosomal–autophagic systems. In diabetes, decreased AKT expression in the skeletal muscle weakens the inhibition of forkhead box O (FOXO) transcription factors, activating the ubiquitin–proteasome system and promoting muscle atrophy [9,10,11].

Persistent hyperglycemia leads to the formation of advanced glycation end-products (AGEs), which interact with their primary receptor, the receptor for advanced glycation end-products (RAGEs), playing a pivotal role in the development of diabetic complications [12,13]. Evidence from several studies indicates that RAGE functionality relies on its ability to form oligomers or heterodimers with other cell surface proteins, such as heparan sulfate proteoglycans, which are essential for the assembly of the oligomeric complex necessary for ligand binding [14,15,16]. The interaction between the extracellular domain of RAGE and its ligands initiates the transcription of proinflammatory cytokines, adhesion molecules, and profibrotic growth factors, collectively driving cellular stress and exacerbating disease-related complications [17,18].

RAGE activation by AGEs was already demonstrated as responsible for the activation of transforming growth factor β (TGF β)/Smad signaling, a profibrotic pathway, in renal tubular cells and liver fibrosis [19].

Resveratrol (RSV), a natural polyphenolic compound, has demonstrated various beneficial effects on cardiovascular, metabolic, and immune functions. In experimental models of obesity and type 2 diabetes, RSV has been shown to improve insulin sensitivity, increase mitochondrial content, and enhance overall survival. While most of RSV’s protective effects are attributed to Sirtuin-1 (SIRT-1) activation, evidence also suggests that it functions through SIRT-1-independent pathways [20,21,22,23,24]. Studies indicate that RSV exerts its effects by inhibiting the mTOR signaling pathway [25]. RSV also activates peroxisome proliferator-activated receptor-α (PPAR-α), a key regulator of the genes involved in fatty acid (FA) oxidation [26]. Its activity is essential for tissues with high energy demands, like cardiac and skeletal muscles, since they rely heavily on FAs as an energy source in severely impaired glucose uptake, as in type 1 diabetes.

Our previous research has shown that RSV significantly mitigates diabetic cardiomyopathy, as it reduces collagen fiber accumulation in cardiac tissue and improves diastolic dysfunction in diabetic rats [27]. This finding further supports RSV’s potential in addressing diabetic complications.

Despite promising effects in many disease models, its clinical effectiveness remains controversial. Some studies have reported significant improvements in health outcomes, while others have failed to find any significant benefits [28,29]. Also, it is challenging to discern its distinct roles across various tissues.

This study aims to investigate the effects of RSV on molecular pathways in an experimental model of diabetes, with a particular focus on its role in mitigating abnormal tissue growth patterns, including cardiac hypertrophy, skeletal muscle atrophy, and adipose tissue atrophy. Observing the different tissue responses, we seek to enhance our understanding of the molecular mechanisms underlying RSV’s protective effects in these metabolically active tissues, which play crucial roles in energy homeostasis.

## 2. Results

### 2.1. Biochemical and Anthropometric Measurements

At the end of the treatment, in the eighth week, glucose serum concentrations were 133 mg/dL, IQ: 118–143 and 128 mg/dL, IQ: 116–136 for C and RC, and 589 mg/dL, IQ: 481–610 and 586, IQ: 547–641 for the D and RD group.

When indexed to body weight, the heart mass and left ventricular dimensions were significantly higher in the diabetic and RSV-treated diabetic groups than in the control groups, confirming the presence of LV hypertrophy in these diabetic rats. Additionally, the gastrocnemius muscle mass was markedly lower in the D and RD groups compared to the C and RC groups, indicating skeletal muscle atrophy (Table 1).

### 2.2. Cardiac Muscle

**Figure 1:**
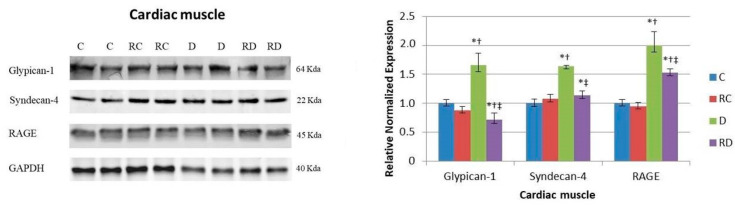
Western blot analysis of Glypican-1, Syndecan-4, and receptor for advanced glycation end-products (RAGE) in cardiac muscle. Control (C), resveratrol control (RC), diabetic (D), and resveratrol diabetic (RD) rats. GAPDH was used to normalize the values. Results are expressed as multiples of control rat results. *p* < 0.05: * vs. C/C; ^†^ vs. RC/C; ^‡^ vs. D/C.

In cardiac muscle (Figure 1), the expression of RAGE was significantly elevated in diabetic rats (D group), and RSV treatment effectively reduced these elevated levels. Among the RAGE cofactors, both Glypican-1 and Syndecan-4 mirrored the RAGE expression pattern, showing a similar increase in diabetic rats and a decrease following RSV treatment.

**Figure 2:**
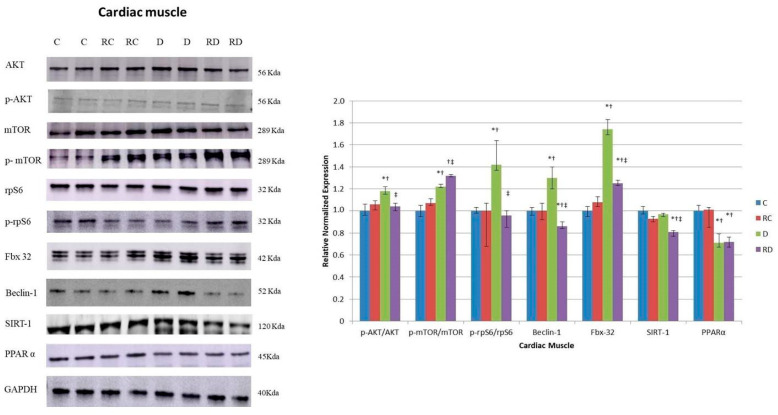
Western blot analysis of protein kinase B (AKT), phosphorylated AKT (p-AKT), mammalian target of rapamycin (mTOR), phosphorylated mTOR (p-mTOR), ribosomal protein S6 (rpS6), phosphorylated rpS6 (p-rpS6), Fbx32, Beclin-1, Sirtuin-1 (SIRT1), and peroxisome proliferator-activated receptor-α (PPARα) levels in cardiac muscle. Control (C), resveratrol control (RC), diabetic (D), and resveratrol diabetic (RD) rats. GAPDH was used to normalize the values. Results are expressed as multiples of control rat results. *p* < 0.05: * vs. C/C; ^†^ vs. RC/C; ^‡^ vs. D/C.

Regarding anabolic proteins, the p-AKT/AKT, p-mTOR/mTOR, and phosphorylated ribosomal protein S6 (p-rpS6)/rpS6 ratios were increased in the diabetic group (Figure 2), suggesting the hyperactivation of anabolic pathways. Following RSV treatment, the concentrations of these proteins decreased, reflecting a potential normalization of protein synthesis regulation. However, the mTOR ratio remained elevated after treatment, indicating a partial restoration of these pathways.

The autophagy-related protein Beclin-1 was also significantly increased in the diabetic group but returned to normal levels after RSV treatment (Figure 2), implying reduced autophagic activity. Fbx-32, a marker of muscle atrophy, was elevated in diabetic rats, but RSV treatment effectively reduced its concentration, suggesting a protective effect against muscle degradation. Lastly, SIRT-1 levels decreased after RSV treatment. PPARα concentrations were reduced in diabetic and RSV-treated groups, indicating that RSV did not reverse the impaired FA metabolism in cardiac tissue under diabetic conditions.

### 2.3. Skeletal Muscle

**Figure 3:**
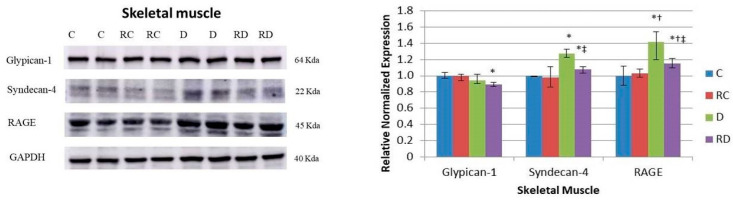
Western blot analysis of Glypican-1, Syndecan-4, and RAGE in skeletal muscle. Control (C), resveratrol control (RC), diabetic (D), and resveratrol diabetic (RD) rats. GAPDH was used to normalize the values. Results are expressed as multiples of control rat results. *p* < 0.05: * vs. C/C; ^†^ vs. RC/C; ^‡^ vs. D/C.

In skeletal muscle (Figure 3), following a pattern similar to that observed in cardiac muscle, RAGE expression was significantly elevated in the diabetic group compared to the control group. This increase was attenuated following treatment with RSV. Syndecan-4, but not Glypican-1, mirrored this trend, further reinforcing the tissue-specific regulation of RAGE and its cofactors in muscle tissues.

**Figure 4:**
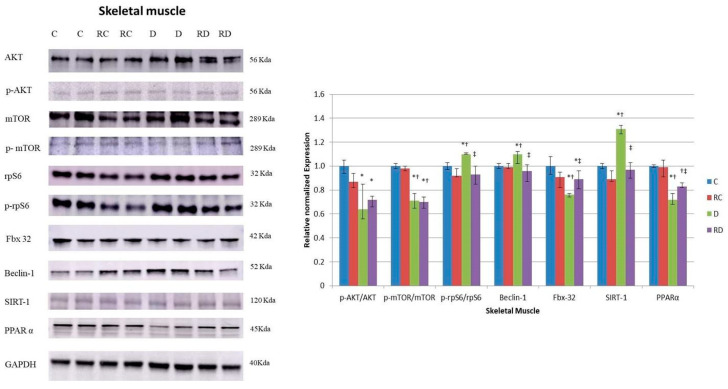
Western blot analysis of p-AKT, AKT, p-mTOR, mTOR, rpS6, prpS6, Fbx32, Beclin-1, SIRT1, and PPARα levels in skeletal muscle. Control (C), resveratrol control (RC), diabetic (D), and resveratrol diabetic (RD) rats. GAPDH was used to normalize the values. Results are expressed as multiples of control rat results. *p* < 0.05: * vs. C/C; ^†^ vs. RC/C; ^‡^ vs. D/C.

Regarding anabolic signaling pathways, the phosphorylation levels of key proteins, including AKT, mTOR, and rpS6, were differentially affected by diabetes and RSV treatment (Figure 4). While the p-rpS6/rpS6 ratio was elevated in diabetic rats and normalized after RSV treatment, the phosphorylation levels of AKT and mTOR remained suppressed in both diabetic and RSV-treated groups, indicating a persistent impairment in anabolic signaling.

Beclin-1, consistent with the findings in cardiac muscle, was increased in diabetic rats and reduced after RSV treatment, suggesting a normalization of autophagic activity. Interestingly, Fbx-32, a marker of muscle atrophy, showed a contrasting response between muscle types. At the same time, its concentration was elevated in the cardiac muscle of diabetic rats, and it was reduced in the skeletal muscle of the same group of rats. No change was observed after RSV treatment, indicating a differential response in muscle atrophy regulation between these tissues. Unlike the cardiac muscle, the skeletal muscle of diabetic rats showed an increase in SIRT-1 levels, which returned to baseline after treatment.

On the other hand, PPARα levels demonstrated a consistent reduction under diabetic and treatment conditions, suggesting disrupted FA metabolism in skeletal muscle, with reduced energy supply from FA. While RSV treatment decreases Beclin-1, a key autophagy protein, tissue wasting persists, suggesting that impaired FA oxidation due to decreased PPARα likely plays an essential role in skeletal muscle loss.

### 2.4. Histopathological Analysis

**Figure 5:**
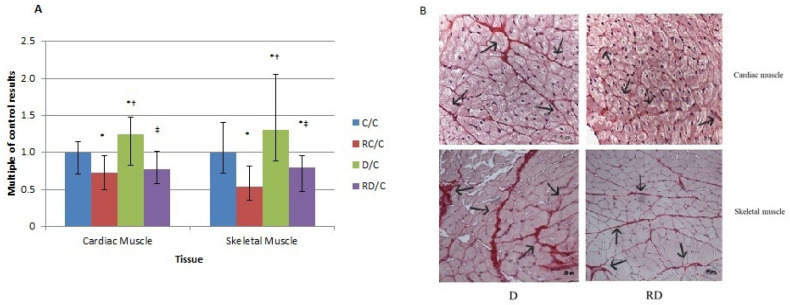
(**A**) Picrosirius staining analysis of cardiac and skeletal muscles. Control (C); resveratrol control (RC); diabetic (D); resveratrol diabetic (RD) rats. Results are expressed as multiples of control rat results. *p* < 0.05: * vs. C/C; ^†^ vs. RC/C; ^‡^ vs. D/C. (**B**) Representative images of Picrosirius staining only for the D and RD groups in cardiac and skeletal muscles. 400×, scale bars: 50 μm.

Picrosirius staining was performed to evaluate collagen content in both cardiac and skeletal muscles (Figure 5). In both muscles, the D group showed a significant increase in collagen, a sign of muscle stiffness, which RSV reversed, indicating less tissue fibrosis.

### 2.5. Adipose Tissue

**Figure 6:**
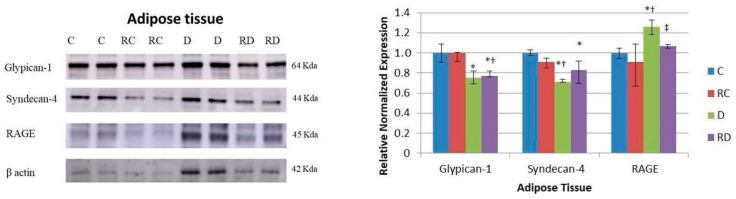
Western blot analysis of Glypican-1, Syndecan-4, and RAGE in adipose tissue. Control (C), resveratrol control (RC), diabetic (D), and resveratrol diabetic (RD) rats. Β-actin was used to normalize the values. Results are expressed as multiples of control rat results. *p* < 0.05: * vs. C/C; ^†^ vs. RC/C; ^‡^ vs. D/C.

In adipose tissue (Figure 6), as in the other tissues, the RAGE level was elevated in diabetic rats, with RSV treatment effectively reducing its expression. However, unlike cardiac and skeletal muscles, neither of the RAGE cofactors, Glypican-1 nor Syndecan-4, followed the same expression pattern, indicating tissue-specific differences in regulating inflammatory pathways.

**Figure 7:**
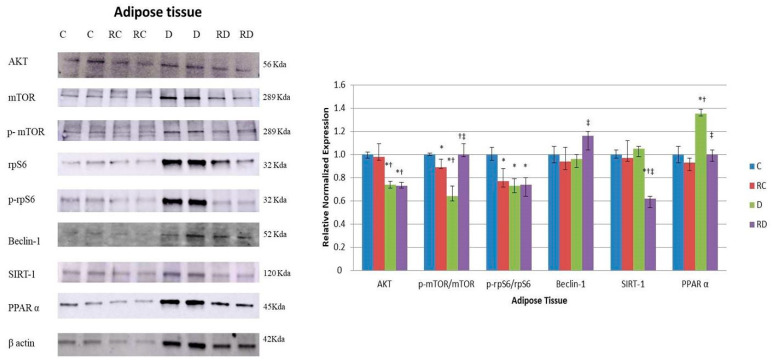
Western blot analysis of p-AKT, AKT, p-mTOR, mTOR, rpS6, prpS6, Fbx32, Beclin-1, SIRT1, and PPARα levels in adipose tissue. Control (C), resveratrol control (RC), diabetic (D), and resveratrol diabetic (RD) rats. GAPDH was used to normalize the values. Results are expressed as multiples of control rat results. *p* < 0.05: * vs. C/C; ^†^ vs. RC/C; ^‡^ vs. D/C.

The expression of anabolic proteins AKT, p-mTOR/mTOR, and p-rpS6/rpS6 was significantly lower in the diabetic group compared to controls, and this downregulation persisted after RSV treatment, except for the p-mTOR/mTOR ratio, which returned to normal levels (Figure 7). This suggests a more profound impairment in protein synthesis in adipose tissue, with RSV treatment having only a partial restorative effect.

**Figure 8:**
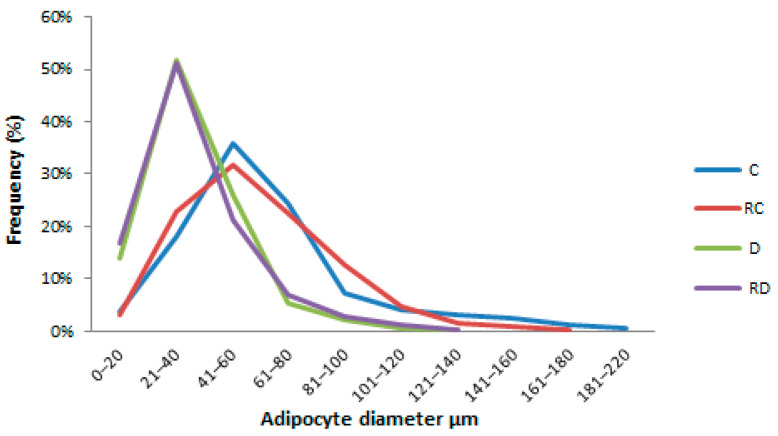
Adipocyte size. Control (C), resveratrol control (RC), diabetic (D), and resveratrol diabetic (RD).

Interestingly, Beclin-1 showed a divergent response in adipose tissue compared to muscle tissues (Figure 7). While its expression was reduced in cardiac and skeletal muscles after RSV treatment, it increased in adipose tissue, indicating enhanced autophagic activity in this tissue following treatment. SIRT-1 levels were decreased after RSV treatment, consistent with the trends observed in muscle tissues. Finally, PPARα concentration was elevated in the diabetic group, which could explain the presence of adipocytes nearly half the size of control cells (Figure 8). Conversely, PPARα was significantly downregulated following RSV treatment.

### 2.6. Adipose Tissue Histopathological Analysis

HE staining of adipose tissue was performed to evaluate adipocyte size in the C, RC, D, and RD groups. Adipocytes in the D and RD groups were smaller (diameter: 34 µm, IQ: 24–45 and 31 µm, IQ: 23–45, respectively) when compared to the C group (61 µm, IQ: 47–79) and RC group (62 µm, IQ: 44–82), with a statistical significance of *p* < 0.0001 (Figure 8).

**Figure 9:**
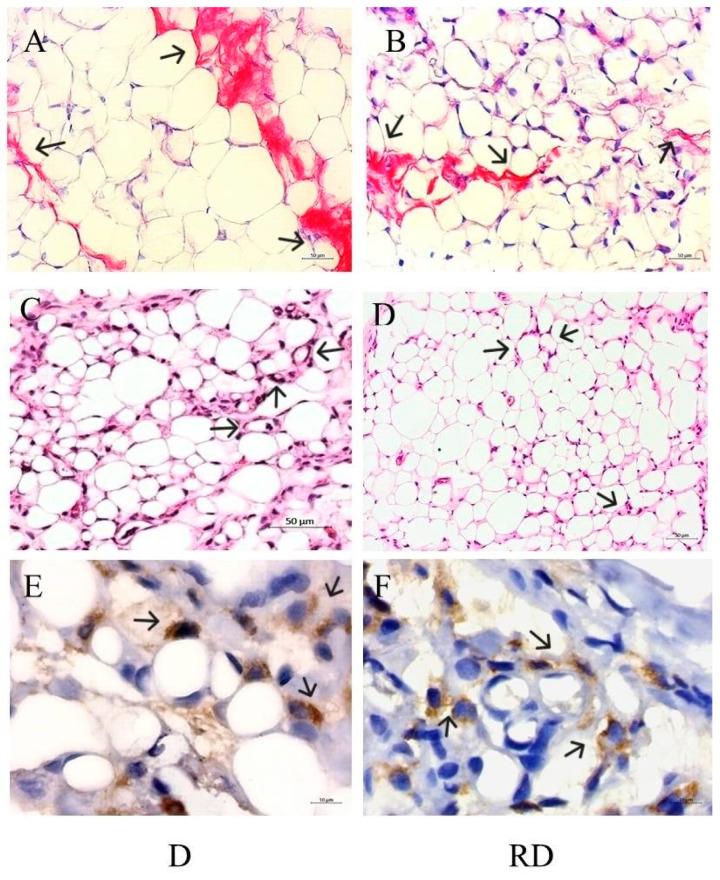
Adipose tissue histological analysis: (**A**,**B**) represent images of picrosirius staining in diabetic (D) and resveratrol diabetic rats (RD). 200×, scale bars 50 µm. (**C**,**D**) represent hematoxylin and eosin staining in D and RD rats. 200×, scale bars 50 µm. (**E**,**F**) represent CD68 immunohistochemistry reactions in D and RD rats. 1000×, scale bars 10 µm. Black arrows indicate collagen staining (**A**,**B**) and crown-like structrures (**C**–**F**).

Picrosirius staining was performed to evaluate collagen content in adipose tissue (Figure 9A,B). According to the results, C/C: 1.00, IQ: 0.45–1.47; RC/C: 0.52, IQ: 0.29–1.35; D/C: 30.15, IQ: 9.66–50.36 vs. RD/C: 8.88, IQ: 4.86–19.06, the D group exhibited a significant increase in collagen accumulation partially mitigated after RSV treatment (*p* < 0.01).

Additionally, macrophage clusters arranged in a crown-like structure surrounding the adipocytes, a marker of inflammation, were identified only in diabetic rats, even after RSV treatment. The presence of macrophages could be detected using HE staining (Figure 9C,D) and by immunohistochemistry reaction using an anti-CD68 antibody (Figure 9E,F).

## 3. Discussion

This study explored the molecular pathways through which resveratrol influences cardiac, skeletal muscle, and adipose tissues in diabetic and non-diabetic rats, comparing their responses to clarify its mechanism of action.

Hyperglycemia-induced inflammation, marked by elevated proinflammatory cytokines, contributes to insulin resistance and promotes advanced glycation end-product (AGE) formation. These AGEs interact with their receptor (RAGE), exacerbating diabetic complications [30,31]. In this study, RAGE levels were elevated in the cardiac muscle of diabetic rats, but RSV treatment reversed this increase. A similar pattern was observed for its cofactors, Syndecan and Glypican.

The D and RD groups showed structural changes in cardiac muscle, including increased heart and left ventricular mass, indicative of the hypertrophic cardiomyopathy commonly associated with diabetes [32]. In our study, RSV treatment reduced collagen accumulation in cardiac muscle tissue. AGE-RAGE interaction stimulates the nuclear factor-kappa B signaling pathway, leading to elevated levels of proinflammatory cytokines such as tumor necrosis factor-alpha. These cytokines are implicated in cardiac hypertrophy, fibrosis, and LV dysfunction pathogenesis. The covalent modification of collagen by AGEs renders it resistant to proteolytic degradation by matrix metalloproteinases, thereby contributing to extracellular matrix accumulation and tissue fibrosis [33]. In cell culture models, RSV inhibited the induction of cardiac fibrosis through the TGF-β1/Smad3 pathway, which attenuates ECM synthesis [34]. Similarly, RSV-induced RAGE downregulation in our study was associated with reduced fibrosis progression. On the other hand, RSV treatment was insufficient to reverse all the tissue structural abnormalities induced by the disease.

Diabetes disrupts the balance between protein synthesis and degradation, leading to muscle hypertrophy or atrophy. Our analysis of anabolic markers, including p-AKT/AKT, p-mTOR/mTOR, and p-rpS6/rpS6, as well as proteins involved in degradation pathways such as the ubiquitin–proteasome system (Fbx32) and the autophagy–lysosome system (Beclin-1), revealed an increase in both anabolic and catabolic protein markers in diabetic cardiac muscle, which is consistent with previous results that demonstrated the activation of the AKT/mTOR/rpS6 axis in hypertrophic cardiac tissue [35]. RSV-mediated inhibition of the key processes of molecular signaling involved in hypertrophic growth may account for our previous findings in which an improvement in cardiac function was observed, characterized by a better diastolic performance [27]. RSV reduced rpS6 phosphorylation, likely through S6K inhibition and apparently without the direct influence of mTOR signaling, highlighting its ability to modulate protein synthesis in diabetic models [36]. A lower presence of Beclin-1 in the RD group was anticipated, as minimal insulin signaling leads to RSV-induced inhibition of S6K1, suppressing autophagy. Considering Beclin-mediated autophagy can be detrimental to the cells, the decrease in Beclin-1 levels caused by RSV could have a protective effect on the cell [37,38].

Similarly to findings in cardiac muscle, the levels of RAGE and its cofactor Syndecan were elevated in the skeletal muscle of diabetic rats and were subsequently downregulated following RSV treatment. RSV also reduced collagen deposition and fibrosis in skeletal muscle; however, it did not prevent muscle atrophy, a common characteristic associated with diabetes [39].

While RSV did not influence the suppressed anabolic markers p-AKT/AKT and p-mTOR/mTOR in atrophic skeletal muscle, it downregulated p-rpS6/rpS6. This effect may be mediated by inhibiting S6K, a pathway linked to suppressed autophagy [37].

As previously noted for cardiac muscle, in skeletal muscle, the elevated level of Beclin-1, a protein linked to catabolism, was reduced after RSV treatment. Although RSV modulated some signaling pathways, it did not reverse the sarcopenia state of the skeletal muscle. One hypothesis is that even though RSV has been capable of protecting against some of the muscle damage caused by diabetes, it could not induce the necessary anabolic response.

Histological analysis showed reduced adipocyte size in diabetic rats compared to controls. In diabetic rats, insulin’s anti-lipolytic effect in adipose tissue is diminished due to lower plasma insulin levels, leading to the breakdown of stored triglycerides into free FAs and glycerol [40]. Simultaneously, insulin’s role in promoting lipogenesis is impaired, reducing fat synthesis and adipocyte storage. The elevated PPARα concentration in diabetic rats further contributes to lipolysis. Together, these factors likely contribute to the observed reduction in adipocyte size.

In adipose tissue, despite a reduction in fibrosis following RSV treatment, the observed atrophic state of the tissue persisted. This phenomenon may share a similar mechanism to that observed in hypertrophic cardiac tissue [34].

Crown-like structures (CLS) were identified within diabetic adipose tissue. These structures, comprising macrophages surrounding dying fat cells, are a sign of inflammation in adipose tissue. These immune cells can release cytokines, attracting additional macrophages to the area and worsening the inflammation [41]. CLS were observed even after RSV treatment in diabetic rats, suggesting that the downregulation of RAGE, a mediator of immune responses, did not change the inflammatory state of the adipose tissue.

Unlike muscle tissues, RSV normalized the reduced mTOR levels found in the adipose tissue of diabetic rats, maybe because mTOR activity is essential for suppressing lipolysis and maintaining systemic lipid homeostasis [42].

The activation promoted by RSV on SIRT-1 is associated with better metabolic processes, reduced oxidative stress, and anti-inflammatory activities [43]. However, instead of an increase in SIRT-1, RSV downregulated its levels in all tissues, suggesting RSV may exert its effects independently of SIRT-1, potentially acting as a competing substrate in protein regulation [23,24].

Cardiac and skeletal muscles and adipose tissue have high energy demands. In conditions of severely impaired glucose uptake, such as in our animal model of diabetes, these systems shift their energy source to FA. FA becomes a primary energy source in skeletal muscle, while in cardiac muscle, where FA is already the primary energy substrate, its consumption increases further. PPARα, a nuclear receptor and transcription factor that regulates fatty acid metabolism, shows increased expression in systems with high FA oxidation rates [44].

In cardiac and skeletal muscles in diabetic rats, PPARα was downregulated potentially as protection against cardiac hypertrophy and due to the chronic inflammatory state associated with diabetes, where elevated cytokines act as inhibitors of PPARα expression [45,46,47]. In our study, RSV treatment failed to restore PPARα levels in these tissues, indicating that hyperglycemic serum concentrations may cause irreversible damage to PPARα expression.

Conversely, in diabetic adipose tissue, PPARα expression is upregulated, likely as an adaptive response to the increased demand for fatty acid (FA) mobilization to meet the energy requirements of other tissues. By restoring PPARα levels to normal, RSV treatment may help reduce lipid storage depletion, potentially preserving adipocyte function.

### Study Limitations

Despite consistent findings, the study’s limitations include the lower dosage of RSV used. Recent research has employed higher concentrations, such as 60, 120, and 240 mg/kg body weight and up to 250 mg/kg body weight, which may elicit more pronounced effects [48,49]. Another area for improvement is the relatively short follow-up period. While this study observed animals for 6 weeks, longer durations, such as 12 and 16 weeks, better capture the cumulative effects of RSV treatment [49,50]. Furthermore, RSV’s efficacy might depend on the progression of type 2 diabetes. It may be more effective in earlier stages when insulin levels remain sufficient to support glucose uptake by cells. In advanced stages, where insulin levels are significantly depleted, the therapeutic benefits of RSV may be diminished.

Based on our findings, future research on RSV treatment should consider alternative models with milder hyperglycemia or include hypoglycemic agents such as metformin. Reducing serum glucose levels may also enable longer-term studies to assess whether RSV induces structural changes in the relevant tissues.

## 4. Materials and Methods

### 4.1. Diabetes Induction

Type 2 diabetes was induced by administering STZ (50 mg/kg IV; Sigma-Aldrich, St. Louis, MO, USA) dissolved in citrate buffer (0.01 M, pH 4.5), followed by an intraperitoneal injection of 100 mg/kg of nicotinamide (Sigma-Aldrich) that was used to attenuate streptozotocin-induced diabetes complications and increase survival rate [51]. The rats were followed for 2 weeks, and at the end of this period, rats with glucose levels ≥ 200 mg/dL were considered diabetics. For 6 weeks, rats from the RC and RD groups received a daily portion (22.04 mg/kg) of RSV (Sigma-Aldrich) diluted in 12% of an aqueous solution of ethanol and administered by gavage, while the control and diabetic groups (C and D) received only 12% ethanol.

### 4.2. Experimental Design

**Figure 10:**
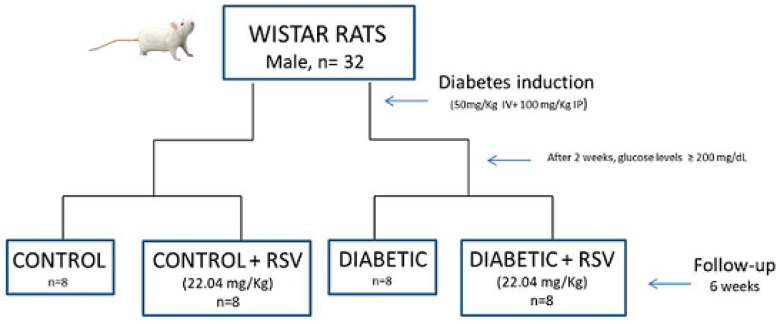
Schematic diagram for study in vivo design. Resveratrol (RSV), intravenous (IV), and intraperitoneal (IP).

Thirty-two Wistar rats (250–350 g) from the University of São Paulo Medical School were randomly assigned to four groups: control (C), control treated with RSV (RC), diabetic (D), and diabetic treated with RSV (RD), with eight animals per group. After 8 weeks, all animals were sacrificed by decapitation. The heart and gastrocnemius muscles were excised, washed in ice-cold 0.9% NaCl, and weighed. Total visceral fat was also collected. Tissues were either prepared for histological analysis, frozen in liquid nitrogen to preserve tissue integrity, and stored at −80 °C (Figure 10). This study adhered to the 2011 Guide for the Care and Use of Laboratory Animals and was approved by the institutional ethics committee.

### 4.3. Muscle Measurements

Heart and gastrocnemius muscle weights were normalized to body mass and expressed as mg/g. Left ventricular mass was also calculated by echocardiogram and normalized to body weight [27].

### 4.4. Histopathological Analysis

For histological analysis, 5 μm sections obtained from formalin-fixed paraffin-embedded cardiac and skeletal muscle and adipose tissue samples were stained with hematoxylin–eosin (HE) and picrosirius red (for collagen detection).

The percentage of positive area stained with picrosirius red (collagen) was evaluated in five randomly chosen histological fields (400× magnification) using the image analysis software ImageJ (1.51J.8, National Institutes of Health, Bethesda, MD, USA). The results were expressed as multiples of the median of the control rats (C), to which a unitary value was attributed.

Adipocyte diameters were measured using HE staining. To obtain representative values, we used the average measurements of adipocyte diameters from five randomly chosen histological fields (all whole cells were evaluated) captured at 200× magnification using the AxioVision system (Zeiss, Oberkochen, Germany). The results were expressed in micrometers as median and interquartile (IQ).

For immunohistochemistry, 5 µm thick sections underwent antigen retrieval in citrate buffer, with a pH of 6.0 for 2 min at 121 psi pressure in a Pascal pan (DAKO Cytomation, Carpinteria, CA, USA). The sections were incubated with anti-CD68 as the primary antibody for macrophage identification (EPR23917, ab283654, from Abcam, Cambridge, UK) and diluted 1:500 in a specific antibody diluent reagent (Thermo Scientific, Waltham, MA, USA). Picture Max kit (Thermo Scientific) was used to detect the antigen/antibody complex. Positive control (rat heart) was treated as described.

### 4.5. Western Blot Analysis

Protein extraction from cardiac and skeletal muscles and adipose tissue was performed using RIPA buffer. Approximately 30 µg of protein per sample was separated by SDS-PAGE and transferred to membranes. Primary antibodies included anti-Glypican-1, anti-Syndecan-4, anti-RAGE, p-AKT/AKT, p-mTOR/mTOR, p-S6rp/rpS6, Beclin 1, Fbx 32, SIRT-1, and PPARα. Protein extraction was performed from the cardiac and skeletal muscle pool obtained from all eight animals of each group using the L-Beader 6 (Loccus Biotecnologia, Cotia, SP, Brazil) tissue disruptor and RIPA buffer (Santa Cruz Biotechnology, Santa Cruz, CA, USA). Protein concentration was determined using the Bradford method. Adipose tissue protein was extracted using the same method, but the tissue was previously pressed between two sheets of filter paper to remove excess fat. Approximately 30 µg of protein were analyzed by 10% SDS-polyacrilamide gel electrophoresis electrotransferred to an Immobilon-P membrane (Millipore, Billerica, MA, USA), blocked and probed with primary antibodies: anti-Glypican-1 (M-95, sc-66909; 1:500), anti-Syndecan-4 (H-140, sc-15350; 1:500), and anti-SIRT-1 (H-300, sc-15404; 1:500), all from Santa Cruz Biotechnology, anti-RAGE (PA1-075; 0,6 µg/mL, Thermo Scientific), mammalian target of rapamycin (mTOR, 7C10; 2983), and the phosphorylated form (p-mTOR; 2971), the ribosomal protein S6 (rpS6, 5G10; 2217) and the phosphorylated form (p-rpS6, 91B2; 4857) (1:1000) from Cell Signalling Technologies (Danvers, MA, USA), p-AKT, AKT (EPR 18853; ab192623 and 16798; ab179463, respectively), Beclin-1 (EPR19662; ab207612), Fbx 32 (EPR914[2]; ab168372), and PPAR α (ab24509) from Abcam (Cambridge, UK) and followed by incubation with secondary horseradish peroxidase (HRP)-conjugated goat anti-rabbit IgG (Calbiochem, EMD Millipore, Burlington, MA, USA, 1:2500). Blots were developed using a Western blot chemiluminescent reagent (Thermo Scientific). The quantification of bands was performed using ImageJ software [52]. A kaleidoscope marker (Bio-Rad, Hercules, CA, USA) was used to measure molecular weights. The pool of each group was run in quadruplicate in two different gels with two samples/gel. Results were normalized by GAPDH (ab9485; Abcam) and beta-actin (sc-130065; Santa Cruz Biotechnology) for adipose tissue values and expressed as multiples of control rat results.

### 4.6. Statistical Analysis

The Kolmogorov–Smirnov normality test was used to analyze the normality of the data. Data were expressed as median and interquartile range (IQR). The comparison between groups was performed using Student’s *t*-test for parametric variables and the Mann–Whitney for non-parametric variables. The significance level adopted for the statistical tests was 5% (*p* < 0.05). Statistical analyses were performed using the MedCalc Statistical Software version 14.12.0 (MedCalc Software bvba, Ostend, Belgium).

## 5. Conclusions

This study highlights the complex tissue-specific responses to diabetes and RSV treatment. RSV treatment selectively influenced protein concentrations in diabetic rats, suggesting that hyperglycemia is a key trigger for its effects.

As we previously reported, RSV effects varied across tissues, with cardiac muscle showing the most significant benefits, including improved cardiac function. This improvement is likely linked to the downregulation of anabolic protein pathways observed in the present study. In contrast to the consistent response in cardiac tissue—where protein expression increased in diabetic rats and decreased after RSV treatment—skeletal muscle and adipose tissue exhibited more heterogeneous responses. Skeletal muscle protein levels, however, showed a trend toward normalization following RSV treatment.

A uniform response across all three tissues in diabetic rats after RSV treatment was a reduction in RAGE production, a key player in diabetic complications, and a decrease in collagen content, leading to reduced fibrosis. In adipose tissue, while RSV lowered RAGE concentration—known for its role in proinflammatory cytokine transcription—it did not reverse inflammation, as evidenced by the crown-like structures in the RD group.

A critical aspect of this study is the persistent and severity of high blood glucose serum concentration in the animals, which may explain why RSV, despite modulating protein expression in key pathways, failed to fully normalize cardiac hypertrophy, persistent sarcopenia in skeletal muscle, and metabolic dysfunction in adipose tissue caused by diabetes.

RSV demonstrates potential in reducing fibrosis and modulating a wide range of protein expressions involved in diabetic complications. Its efficacy likely depends on factors such as plasma concentration, treatment duration, and the severity of hyperglycemia.

## Figures and Tables

**Table 1:** Anthropometric measurements for control (C), resveratrol control (RC), diabetes (D), and resveratrol diabetes (RD) groups obtained at the end of the study.

	C	RC	D	RD
Heart mass/BW (mg/g)	3.22 (2.74–3.51)	3.30 (3.17–3.53)	3.96 *^,†^ (3.57–4.11)	3.94 * (3.43–5.20)
LV mass index/BW (mg/g)	1.87 (1.80–2.00)	1.99 (1.80–2.20)	2.36 * (2.07–2.71)	2.30 * (2.20–2.50)
Gastrocnemius mass/BW (g/g)	5.44 (5.14–5.64)	5.01 (4.84–5.35)	4.4 *^,†^ (4.22–4.54)	4.21 *^,†^ (3.84–4.58)

BW, body weight; LV, left ventricle. *n* = 8. Values are presented as median and interquartile (25th and 75th). *p* < 0.05 * vs. C; ^†^ vs. RC.

## Data Availability

Data is contained within the article.

## Abbreviations

AGE	Advanced glycation end-products
AKT	Protein kinase B
FOXO	Forkhead box O
IGF	Insulin growth factor
mTOR	Mammalian target of rapamycin activity
PPAR α	Peroxisome proliferator-activated receptor alpha)
rpS6	Ribosomic protein S6
SIRT-1	Sirtuin 1
TGFβ	Transforming growth factor β
RSV	Resveratrol
